# South Africa

**Published:** 1900-02-03

**Authors:** 


### SOUTH AFRICA.

It has been a common experience in English warfare that the supreme hour has brought the man. History is full of examples. Cromwell, Marlborough, Wolfe, Clive, Wellington, Clyde, Havelock, all appeared when there was urgent need of them, and it can hardly be doubted that the same will happen again. We must wait and hope; and, in the meanwhile, it will be a wholesome exercise to fix our thoughts upon the few bright spots which appear in the otherwise dismal landscape. One of these bright spots is the splendid bravery and conduct of the regimental officers and of the rank and file, which shows that our race has not degenerated; another, and one of the most conspicuous undoubtedly, is supplied by the devotion to duty of the officers of the Army Medical Corps, as well as by the admirable skill and the masterly organisation with which this devotion has been utilised and directed. In reading the letter written after the battle of Colenso by Mr. Treves, and extensively republished, we are almost tempted to forget the ghastly carnage in our admiration of the care which was taken to minimise the sufferings of the wounded. No one who has not witnessed such a scene can possibly form any adequate conception of it, and no one who has not shared in the responsibility of assisting to diminish and control its horrors can form any adequate conception of the strain which it would cast upon the energies of the officers in charge. "The men," writes Mr. Treves, "were coming in as fast as the ambulances and bearers could bring them. Some were dead, some were dying, all were parched with thirst, and baked and blistered with heat. The men were lying on all sides on stretchers, amidst tents, piles of rifles, accoutrements, battered helmets, and blood-stained tunics; and the blazing sun added to the miseries of all." In such a scene as this, with everything to excite the most painful emotions, the officers of the Medical Corps had to keep their heads cool and clear, to avoid the expenditure of their precious time upon cases in which hopelessness was recognisable, to select with rapid glance those in which immediate care was essential to success, to pass over for the moment those who would be the least liable to suffer from delay, and, uninfluenced by the horrors of the scene, to decide with unhesitating rapidity, and to act with unfailing promptitude. Towards the discharge of such duties they had every help which organisation could afford; and the existence of this organisation, as well as the necessity for its being kept under the control of strict and steady discipline, are sufficient to establish beyond the possibility of cavil the soundness of the claim for the exercise of military command which has been so strenuously and pertinaciously urged on behalf of medical officers, and long so stubbornly resisted by those in authority. Colonel Gallwey has had his work to do, and he has done it in a way which must obtain the unstinted admiration of all who read its record. He had obtained a volunteer ambulance corps of 2,000 men, of whom 12 were allotted to each stretcher, with the general result that the field was cleared of wounded on the evening of the battle, and that 800 of them were attended to by 16 surgeons. Nor must we omit to mention Major Brazier-Creagh's admirable hospital train, in which those of the wounded who could be moved were at once conveyed, with every possible care and comfort, either to Estcourt or to Maritzburg; or the pluck of Major Baptie, who rode under a hail of bullets into a donga to succour the wounded, whose horse was shot under him as he went, and who remained with them, dealing out teaspoonfuls from his solitary water-bottle until the fight was over. Mr. Treves rightly says that his conduct calls for some recognition from the medical profession, "if not from the military authorities."

